# The Umbilical Cord Clamp Method—Procedural Description and Safety Assessment of a Novel Method of Umbilical Catheter Fixation After Side Entry Insertion

**DOI:** 10.3390/life15121935

**Published:** 2025-12-18

**Authors:** Anna Tomaszkiewicz, Piotr Kruczek, Piotr Szymański, Piotr Teplicki, Rita Abu Faraj-Batko, Alina Sobczak, Sonia Kahtan, Boris W. Kramer, Jan Mazela

**Affiliations:** 1 Department of Neonatology, Poznan University of Medical Sciences, Fredry St. 10, 61-701 Poznań, Polandjanco@pol-med.com.pl (J.M.); 2 Department of Neonatology, R. Czerwiakowski Gynecology and Obstetrics Hospital, Siemiradzkiego St. 1, 31-137 Kraków, Poland; kruczekpiotr@poczta.onet.pl (P.K.);

**Keywords:** umbilical venous catheter, umbilical catheter, neonatal intensive care, catheter-related complications, ultrasound, catheter fixation, catheter migration

## Abstract

**Background:** Umbilical venous catheter (UVC) placement is common in neonates but carries risks of migration and infection. This study evaluates safety of a novel fixation technique using the umbilical cord clamp after a side-entry insertion. **Methods:** A retrospective analysis of 264 neonates was conducted at a tertiary center in order to assess safety of the novel UVC fixation method. The new technique involved side-entry catheter insertion without severing the cord, secured to the clamp with a sterile patch. Catheter tip position was confirmed and monitored every 24 h via ultrasound. **Results:** Catheter migration occurred in 18.9% of cases, mostly inward into the right atrium which was managed by repositioning. Migration into the ductus venosus requiring removal occurred in 0.7% of cases and unscheduled removal due to stump detachment in 1.5%. No central line-associated bloodstream infections (CLABSIs) were observed. **Conclusions:** The umbilical cord clamp method is a safe, single-operator alternative for UVC fixation. This technique had a low rate of catheter migration, did not increase the risk of infection, and was cost-effective and simple.

## 1. Introduction

Placement of umbilical catheters in children is one of the most common procedures performed for vascular access in newborns in neonatal intensive care units.

The standard and commonly accepted method of inserting a UVC involves grasping the cord with the artery forceps and pulling the cord upwards, which is performed by one person, while the second operator cuts the umbilical cord underneath. Then, after identification of the vessels, the catheter is inserted into the umbilical vein or artery. Finally, the catheter should be attached, which is most often performed by sewing the catheter to the umbilical cord [1,2] and/or by creating a tape bridge between the catheter and the abdominal wall [3].

A key challenge with UVC placement is catheter migration [4,5], which can lead to complications or complete catheter dislodgement. Previous studies report UVC malposition rates as high as 74% [4,5,6]. Another significant concern is the risk of infection, which can arise from both the insertion procedure and prolonged catheter dwell time.

This study evaluates a modified approach to UVC placement, referred to as the umbilical cord clamp method. In this technique, the cord clamp is not removed prior to inserting the umbilical catheters, and vascular access is obtained via a side-entry incision. Retaining the clamp allows the operator to stabilize the umbilical stump manually during catheter insertion without the need for additional instruments. In practice, this enables the operator to hold the stump with one hand while advancing the catheter into the vessel lumen with the other. A further advantage of leaving the clamp in place is that it can later be used to secure and protect the catheters once they have been inserted. This configuration also enables the procedure to be performed by a single clinician, which represents an additional benefit of the method.

The primary aim was to assess the technique’s impact on catheter migration and CLABSIs. Additionally, we examined the risk of unplanned catheter removal associated with this technique.

## 2. Materials and Methods

### 2.1. Study Design and Patient Population

We conducted a retrospective study at the tertiary-referral neonatal center from 1 July 2023 to 1 January 2024. The study was approved by Poznan University of Medical Sciences Bioethics Committee, Poland. Medical records of 268 neonates requiring UVC placement per institutional protocol were reviewed. Inclusion criteria were the need for parenteral nutrition, fluid therapy or medication administration. Exclusion criteria were loss of catheter supervision due to patient transfer or other reasons. In summary 264 patients were included in the final analysis.

### 2.2. The Umbilical Cord Clamp Method

**Figure 1:**
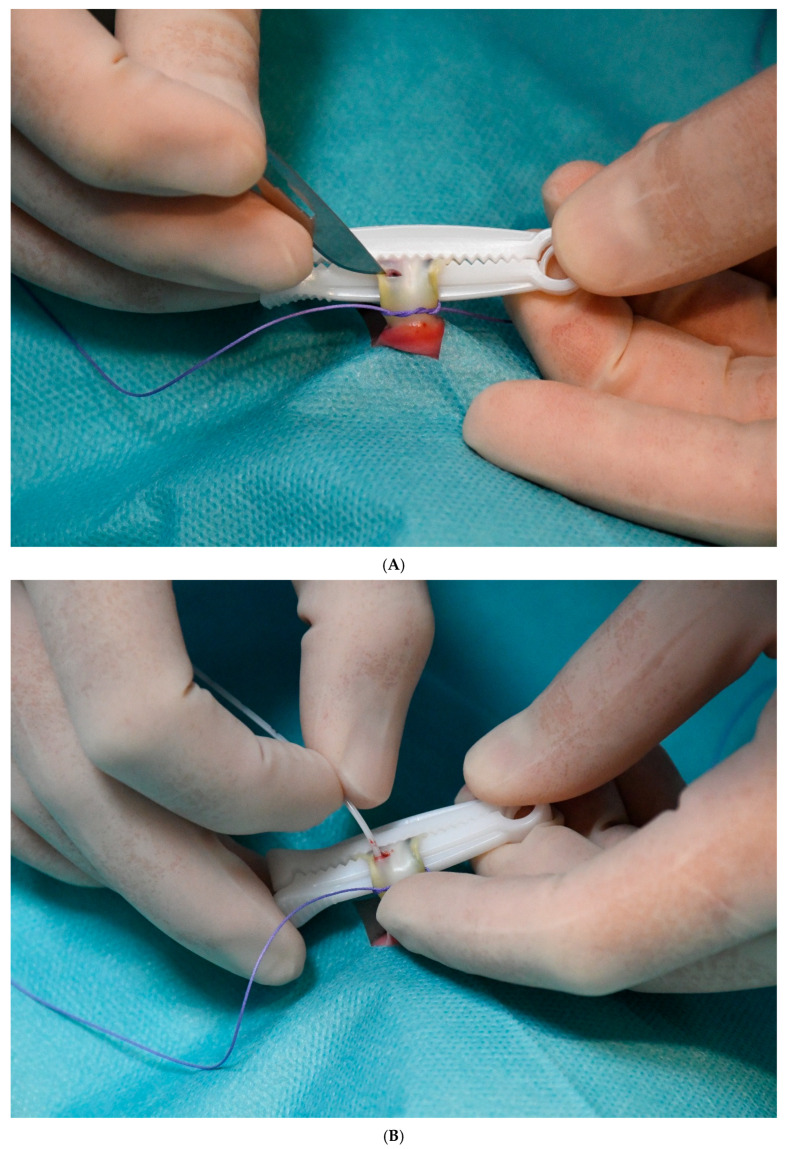

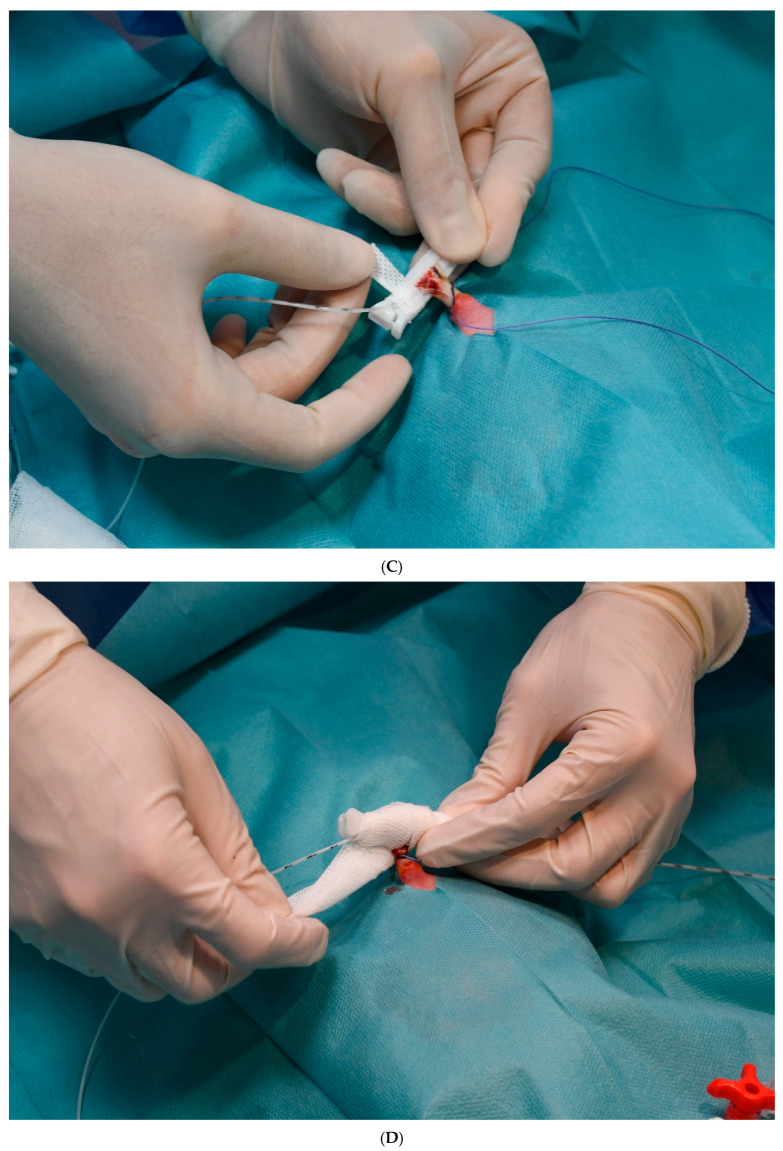
(**A**) Stages of UVC fixation using the umbilical cord clamp method. A Transverse incision of the umbilical vein (“side entry”) after identifying the vein, which is visible through the umbilical cord tissue. (**B**) Insertion of the catheter into the lumen of the umbilical vein. (**C**) Fixation of the catheter to the umbilical cord clamp with a sterile patch after confirming correct catheter position on ultrasound. (**D**) Wrapping of the umbilical cord clamp and catheters with sterile gauze.

The initial stage of umbilical catheter insertion is comparable to the conventional method, involving thorough cleansing and disinfection of the umbilical cord and the umbilical clamp, followed by sterile draping of the peri-umbilical region and the application of umbilical tape. Octenidine dihydrochloride was used for disinfection. An umbilical cord clamp (Intergos, Bielsko-Biała, Poland) was employed. In contrast to the standard method, the umbilical cord stump with the umbilical cord clamp is not cut off. This constitutes the principal modification, in addition to the side-entry technique. The umbilical cord is then carefully examined, and the sites where the umbilical vessels are visible in the longitudinal axis are identified. Once the umbilical vein has been located, a transverse incision is made and the anterior wall of this vessel is gently incised just below the umbilical cord clamp (“side-entry”; Figure 1A), which is positioned approximately 1 cm from the ventral attachment of the umbilical cord. A catheter is subsequently inserted into the vessel lumen (Figure 1B).

A 3.5-Fr gauge Vygon polyurethane single-lumen catheter is used. The catheter is advanced to the correct position under ultrasound guidance. The ultrasound probe is placed beneath the sterile drape and does not come into contact with the operative field. The catheter tip is positioned in the subdiaphragmatic vestibule. Correct tip placement is determined exclusively by ultrasonography, in accordance with the neonatal DAV-expert algorithm [7]. Once correctly positioned, the catheter is secured firmly with a sterile patch to the umbilical cord clamp (Figure 1C).

The final step in the procedure is to wrap the umbilical cord clamp and catheter with sterile gauze (Figure 1D), thereby protecting the catheter from the external environment.

**Figure 2:**
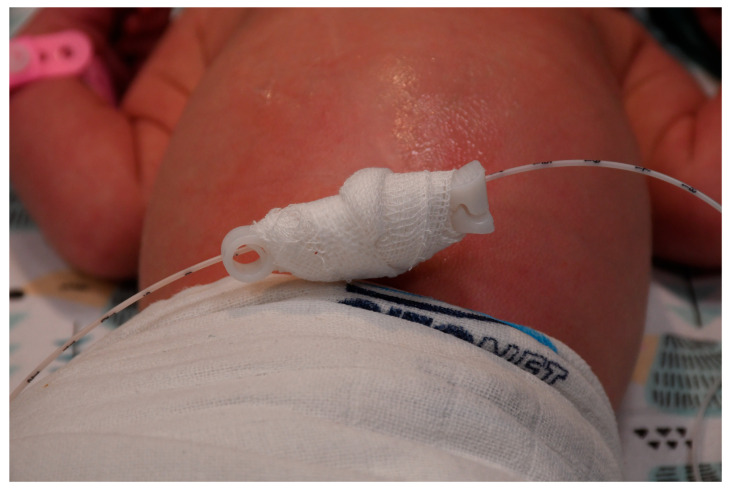
Patient with UVC and UAC placed using the umbilical cord clamp method.

If the external dressing becomes contaminated, for example, with blood, it is replaced with a new one. Figure 2 shows a patient with an umbilical arterial catheter (UAC) and an umbilical venous catheter (UVC), both inserted using the umbilical cord clamp method.

The procedure can be performed by a single clinician, which represents another significant advantage of this method. In practice, one operator manages the entire catheterization, while a second clinician participates only during catheter advancement under ultrasound guidance. Both the operator and the operator of the ultrasound machine are either specialists or trained residents. A trained resident is defined as an individual who has performed at least 25 procedures and has participated for a minimum of two months in observing catheter placement and maintenance in neonates.

### 2.3. Outcomes

Our primary endpoint were catheter tip migration and catheter discontinuation due to malposition.

The secondary outcome was the number of CLABSIs. Since in the method described, the catheter must be attached to the umbilical clamp, in the study we also examined whether there was unplanned catheter removal associated with the umbilical stump falling off together with the clamp and catheter.

Catheter tip migration was defined as displacement from the optimal position—that is, from the subdiaphragmatic vestibule—either inward (too deep, into the cardiac chambers) or outward into shallower locations such as the ductus venosus or portal venous system.

In cases of inward migration (catheter too deep in the heart), the catheter was repositioned under ultrasound guidance. Outward migration (catheter too shallow) required catheter removal.

CLABSI was defined as the recovery of a pathogen from a blood culture in a patient who had a UVC at the time of infection or within 48 h before the development of infection.

Unscheduled catheter removal was defined as the detachment of the umbilical catheter alone or with stump and clamp.

### 2.4. Evaluation of Catheter Tip Displacement

**Figure 3:**
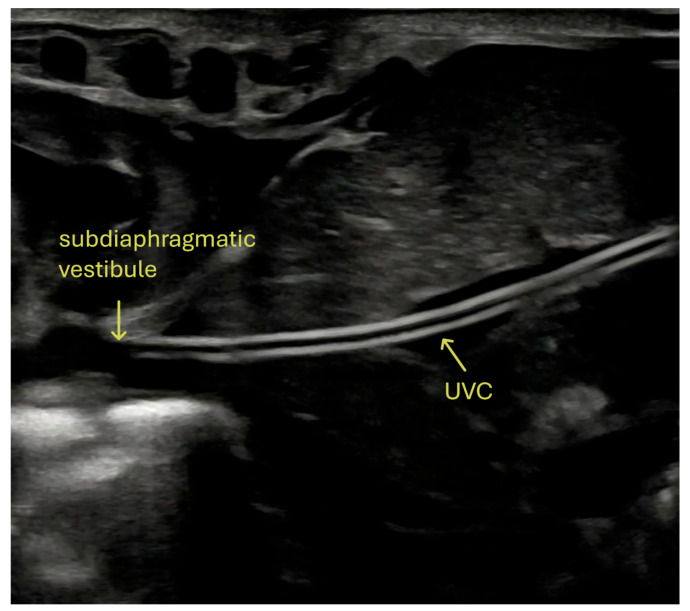
Ultrasound-guided positioning and monitoring of the UVC in the correct subdiaphragmatic vestibule location.

Ultrasonography was used to assess catheter tip displacement. All examinations were performed by ultrasonographers with extensive experience in the evaluation of central catheters and certified by the Polish Ultrasound Society, confirming their competence in neonatal ultrasonography. The study was supervised by a senior ultrasonographer with 30 years of experience in ultrasound imaging and more than a decade of experience in assessing vascular catheters. In cases of uncertainty, the supervising specialist reviewed and confirmed the findings. All ultrasound examinations were recorded. Ultrasound examinations were performed using a 12–15 MHz linear probe on Philips HD15, Philips Affiniti (Eindhoven, The Netherlands), or Samsung V8 ultrasound scanners (Seoul, Republic of Korea). The catheter was monitored by ultrasound every 24 h to assess tip position and potential complications (Figure 3).

### 2.5. Statistics

The calculations were performed using Statistica 13 software from TIBCO and PQStat 1.8.6 software from PQStat. The significance level was set at alpha = 0.05. The result was considered statistically significant when *p* < alpha. The normality of the distribution of variables was tested using the Shapiro–Wilk test, and the equality of variances was tested using Levene’s test. In order to compare variables between the two groups, in the case of normal distribution and equal variances, the unpaired t-test was used, and in the case of non-normal distribution, the Mann–Whitney test was used. In order to examine the relationship between categorical variables, the chi-square independence test, Fisher’s exact test or Fisher-Freeman-Halton test were calculated. In the case of categorical data comparison, when Cochran’s condition regarding the expected number was met, the chi-square independence test was used, and when it was not met, Fisher’s exact test (for 2 groups) or Fisher-Freeman-Halton test (for more than 2 groups) was used.

## 3. Results

**Figure 4:**
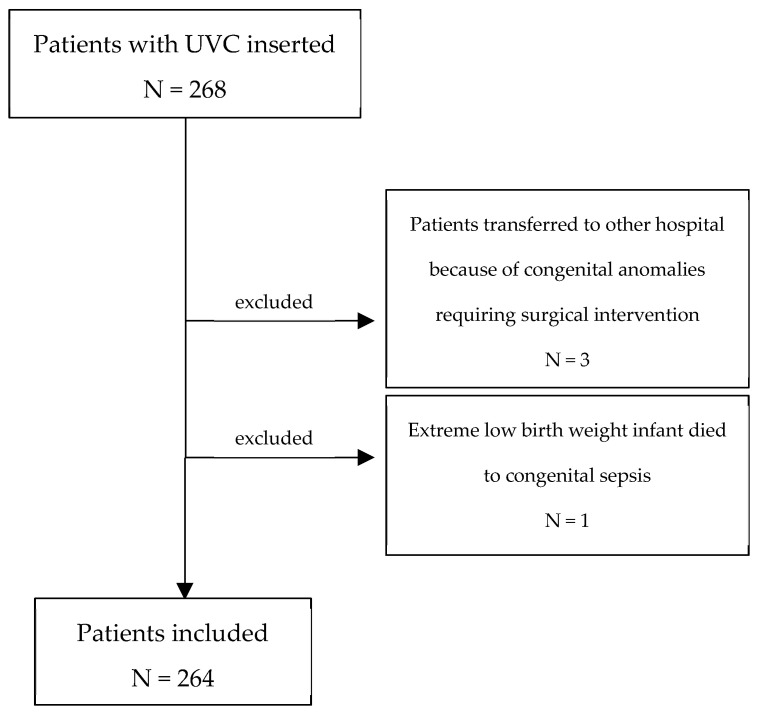
Flow chart of study population detailing included and excluded patients.

Of the 268 patients, three neonates were transferred to other hospitals within 24 h because of congenital anomalies requiring surgical intervention, and one extremely preterm neonate, born at 24 weeks of gestation, died within the same period due to congenital sepsis (Figure 4). In the case of 7 patients, data on migration was missing (2.65%).

All catheters were inserted within 24 h after birth. Catheter tip migration occurred in 18.9% (50/264) of cases. Two catheters (0.7%) migrated into the ductus venosus, which required immediate removal. In 18.1% (48/264) of cases, the catheter tip migrated deeper into the right atrium but was repositioned under ultrasound guidance and subsequently removed as planned when no longer medically required.

Most catheters that migrated and required repositioning (48 catheters) did so on the second day (25 patients). On the day of insertion, 7 catheters were repositioned. On the third day, 11 catheters were repositioned, followed by 4 catheters on the fourth day and 1 catheter on the sixth day.

The analysis found that patients who experienced migration had a statistically significantly lower mean gestational age, longer hospital stay, and longer catheter dwell time (Table 1). No differences were found between the migration and non-migration groups in terms of indications for catheterization, presence of UAC, and gender. Detailed results are presented in Table 1.

Unscheduled catheter removal due to the umbilical stump detaching with the clamp occurred in 1.5% (4/264) of cases. No statistical significance was found between children who experienced this type of complication and children without this complication in relation to factors such as gestational age (*p* = 0.42), birth weight (*p* = 0.71), catheter dwell time, (*p* = 0.67), length of hospital stay (*p* = 0.42), presence of UAC (*p* = 1.0), and gender (*p* = 0.32).

No cases of CLABSI were observed.

One neonate with a deeply positioned catheter developed a pericardial effusion, which resolved spontaneously after repositioning.

## 4. Discussion

This is the first study to describe a modified method of fixing umbilical catheters to the umbilical cord clamp, offering a potential alternative to the standard catheter fixation technique.

A theoretical concern with this method is the risk of infection, as the catheter is secured using a sterile patch while the umbilical cord clamp itself is not sterile, despite meticulous disinfection prior to the procedure. There are multiple factors that may influence the incidence of CLABSI in neonates. One of the most frequently discussed is dwell time, although not all studies confirm a direct association. For instance, the randomized controlled trial by O’Hara et al. failed to demonstrate such a correlation [8]. In a comprehensive review, Corso summarized studies investigating the relationship between UVC dwell time and CLABSI incidence, aiming to determine the optimal duration for catheter removal or replacement with a peripherally inserted central catheter, thereby minimizing infection risk [9]. Reported CLABSI rates across the studies reviewed vary widely - from 1 CLABSI per 1000 catheter-days to 42 per 1000 UVC-days - reflecting differences in study design and patient populations. In our study, the mean dwell time for the entire cohort was five days, which we consider sufficient to assess whether infection occurred during catheter insertion, as this period allows for the clinical manifestation of catheter-related infection.

However, our findings do not support an increased infection risk associated with this approach.

Catheter migration is another significant issue associated with UVC placement, as it can lead to severe complications. Catheter malposition can result in life-threatening consequences, including bleeding and necrotizing enterocolitis in infants with catheter tips displaced into the ductus venosus [10]. Case reports have also described liver abscesses [11,12] and cardiac tamponade [13,14,15].

It has been proposed that UVC tips may move as postnatal anatomy evolves. Cord contraction can draw the tip inwards, and increases in lung volume—especially after surfactant—may displace the target area caudally. Later outward migration is thought to result from gradual abdominal distension as the bowel fills with gas [5]. In our study, catheter tip migration occurred in only 18.9% of cases, with most displacements being inward. Importantly, no severe adverse effects such as NEC or bleeding were observed, apart from one case of pericardial effusion that resolved spontaneously without hemodynamic consequences.

In all cases of inward displacement, the catheter was repositioned under ultrasound guidance without requiring removal. The clamp method simplifies this repositioning process by allowing clinicians to easily adjust the catheter after removing the external dressing and sterile patch (see Figure 1A,B).

Catheters were removed in two cases (0.7%) due to displacement into the ductus venosus, a rate substantially lower than previously reported. In the study by Franta et al. [16], 2 of 65 infants (3%) had catheters positioned within the liver at the first ultrasound assessment. Anneloes M. Plooij-Lusthusz reported that 25% of catheters were placed too low [17], while in the study by Gerdina H. Dubbink-Verheij, 14% of catheters assessed during ultrasound monitoring were located too low, and 2% were abnormally low [4]. The highest migration rate was described by Hoellering et al. [5], with 55% of catheters found to be too low at ultrasound assessment prior to removal. It is worth emphasizing that in our study, no catheter was positioned below the level of the ductus venosus. This low rate of catheter loss demonstrates the effectiveness of the clamp method in minimizing malposition-related discontinuations.

Centorrino et al. recommended the use of ultrasound for catheter tip localization, both during the procedure and for subsequent monitoring, in view of the high frequency of catheter migration [18]. This strategy was adopted in our unit. We did not use X-rays to assess the position of the catheters.

In Table 2, we summarize the key differences between the umbilical cord clamp method and the standard technique, along with the outcomes of our study. It should be noted that the reported rates of complications vary considerably across studies, as discussed earlier in the manuscript.

Alternative methods for securing UVCs have been investigated in recent years. One such method resembles the ‘side-entry method’ described over 35 years ago by Squire et al. [19]. The similarity lies in the side incision of the umbilical vessel without severing the cord, and in the fact that only one doctor is required to perform the procedure. However, it differs from the umbilical cord clamp method in the way the catheter is secured. The ability to insert a catheter without the need for a second doctor is particularly valuable, especially in emergency situations. Both the side-entry method and the umbilical cord clamp method offer this advantage.

Rüdiger et al. [20] emphasized in a letter to the editor that they had successfully applied the side-entry method at their institution for many years. Similarly, the clamp method has been used locally in Kraków, Poland, for many years by some neonatal units, but it has not previously been described in the literature, nor has its safety regarding infection or its effectiveness in preventing catheter migration been studied. This paper aims to address these gaps for the first time.

Other alternative methods for UVC fixation have also been explored. D’Andrea et al. [21] evaluated the use of cyanoacrylate glue to reduce catheter dislodgement. In this method catheters were secured with a suture supplemented by four drops of octyl-butyl-cyanoacrylate adhesive. A 3/0 silk suture was placed through the cord tissue (avoiding skin and other vessels) and loosely tied so it could be wrapped around the catheter two or three more times. The final knot prevented slippage while still allowing unobstructed infusion and aspiration [21]. Alternative technique was used by Perl et al. [22] with a device called LifeBubble to minimize catheter migration. LifeBubble is a securement device designed for umbilical catheters, which is a small sterile silicone dome with a cleat and strap that adheres to the infant’s abdomen allowing ventilation and visibility of the catheters and depth markers while shielding the site.

Both approaches demonstrated lower migration rates compared to our findings. However, in those studies, higher rates of discontinuation due to malposition were reported. Furthermore, Perl et al. [22] relied on X-rays to assess catheter displacement, whereas our study uses ultrasound, which provides a more sensitive and precise assessment of catheter tip position [23,24]. Unlike these alternative methods, the umbilical cord clamp method does not require additional devices or materials beyond standard NICU equipment. Our results suggest that this method offers comparable benefits without the need for specialized resources, making it a practical and cost-effective option for UVC fixation. It is also worth noting that in both the LifeBubble technique and the umbilical cord clamp method, the catheter entry site is protected, unlike in the standard method, where the site remains exposed to external factors.

One limitation of the umbilical cord clamp method is the natural process of umbilical cord shrinkage and detachment, which may lead to unscheduled catheter removal. In this study, removals due to the umbilical stump detaching with the clamp occurred in 1.5% of cases, which highlights a potential limitation of the technique. On the other hand, the natural drying of umbilical stump appears to provide an effective stabilizer for the UVC, ensuring fixed positioning.

A limitation of our study is its retrospective design. In addition, the study population primarily consisted of late preterm and term infants with respiratory failure. Future prospective studies should include a larger cohort of extremely preterm infants to further evaluate the safety and effectiveness of this method, especially since statistical analysis has shown that the risk of migration is higher in children with lower gestational age and lower body weight. Our study was conducted at a single center, which may affect its validity, as the clinicians inserting the catheters were already highly familiar with the technique and had used it routinely for many years. The ultrasound training of the medical staff at this institution is also above average. Nevertheless, the insertion technique itself appears straightforward and does not require additional instruments, suggesting that the learning curve in other centers would likely be short.

## 5. Conclusions

The umbilical cord clamp method proved to be a simple, single-operator technique associated with a low rate of catheter migration and no observed CLABSI. Most migrations were inward and could be safely corrected under ultrasound guidance, with only two catheters requiring removal due to displacement into the ductus venosus. These findings indicate that the method is a feasible and cost-effective alternative to standard UVC fixation, although prospective studies in more vulnerable populations are warranted.

## Figures and Tables

**Table 1:** Comparison of patients with catheter migration and patients without catheter migration.

Variable	Patients with Migration	Patients Without Migration	*p* Value	Effect Size
Gestational age (median)	36	37	0.046	0.100 **
Birth weight (grams)	2393.7	2840.76	0.0004	0.614 *
Length of hospital stay (days)	19	12	0.0003	−0.205
Catheter dwell time (days)	6.2	4.7	0.0001	−0.227
Female sex	44%	44.40%	0.95	
UAC presence	68.60%	76%	0.31	
Primary Indications for Catheterization:				
Prematurity	34%	34.30%	0.83	
Respiratory Failure	26%	26.09%		
Low Birth Weight	16%	11.11%		
Hypoglycemia	8%	8.21%		
Congenital Malformations	2%	4.83%		
Asphyxia	0%	3.86%		
Infection	4%	2.42%		
Other	10%	9.18%		

*—Cohen’s d, **—r effect size.

**Table 2:** Basic differences between the standard and the umbilical cord clamp methods and their complications.

Variable	The Umbilical Cord Clamp Method	Standard Method
**Migration**	18.1% *	Up to 74%
**CLABSI**	0 *	Up to 42 per 1000 UVC-days
**Operator**	Single	Two operators
**Tip confirmation**	Ultrasound	X-ray or ultrasound
**Umbilical catheter fixation**	Sterile patch to umbilical cord clamp	Sutures
**Correction of catheter position**	Easy	Difficult
**Umbilical catheter site**	Protected by sterile gauze	Unprotected

* in our study.

## Data Availability

The source data is available from the corresponding author on reasonable request.

## Abbreviations

The following abbreviations are used in this manuscript:

UAC	Umbilical Artery Catheter
NICU	Neonatal Intensive Care Unit
CLABSI	Central Line Confirmed Blood Stream Infection
